# Raising awareness of *Demodex mites*: a neglected cause of skin disease

**DOI:** 10.1007/s15010-025-02521-z

**Published:** 2025-05-03

**Authors:** Amal El-Moamly, Omar El-Swify

**Affiliations:** 1 Department of Medical Parasitology, Faculty of Medicine, Suez Canal University, Round Road, Ismailia, 41522 Egypt; 2 Medical Services Department, Suez Canal University, Ismailia, Egypt

**Keywords:** *Demodex*, *Folliculorum*, *Brevis*, Arachnida, Mite, Demodicosis, Neglected disease, Skin, Eye, Opportunistic infection, Microbiome, Pathogenicity, Clinical manifestations, Diagnosis, Treatment, Future prospects

## Abstract

**Background:**

*Demodex* mites are among the most prevalent human parasites. While commonly found on healthy individuals, an overpopulation of this arachnid resident of human skin triggers demodicosis, a neglected yet widely prevalent disease with considerable skin and eye morbidity. Despite its health impact, demodicosis remains overshadowed by other common skin diseases. This neglect has significant consequences for individual and public health, which require a paradigm shift in our understanding and management of this ubiquitous ectoparasite.

**Main abstract body:**

We reviewed the literature to re-evaluate the pathogenicity of the *Demodex* mite, paying particular attention to the primary risk factors—immune dysregulation, altered microbiota, and concurrent infections—that may contribute to pathogenicity. We discuss the challenges in combating neglect of demodicosis and provide updates on various impediments in achieving this goal. We explore the issues and research gaps in various domains such as those related to parasite biology, pathogenesis, diagnosis, treatment, prevention and control. We present potential solutions and outline future prospects for tackling this important disease. Finally, we hope to catalyze greater attention and investment for this neglected public health issue.

**Conclusion:**

Raising awareness of *Demodex* and demodicosis and its major contribution to human diseases requires a multidisciplinary approach. Efforts to prioritize its place on the global health agenda, invest in research, improve diagnostic tools, and develop new treatment strategies will lead to improved public health outcomes and a higher quality of life for those affected.

**Graphical abstract:**

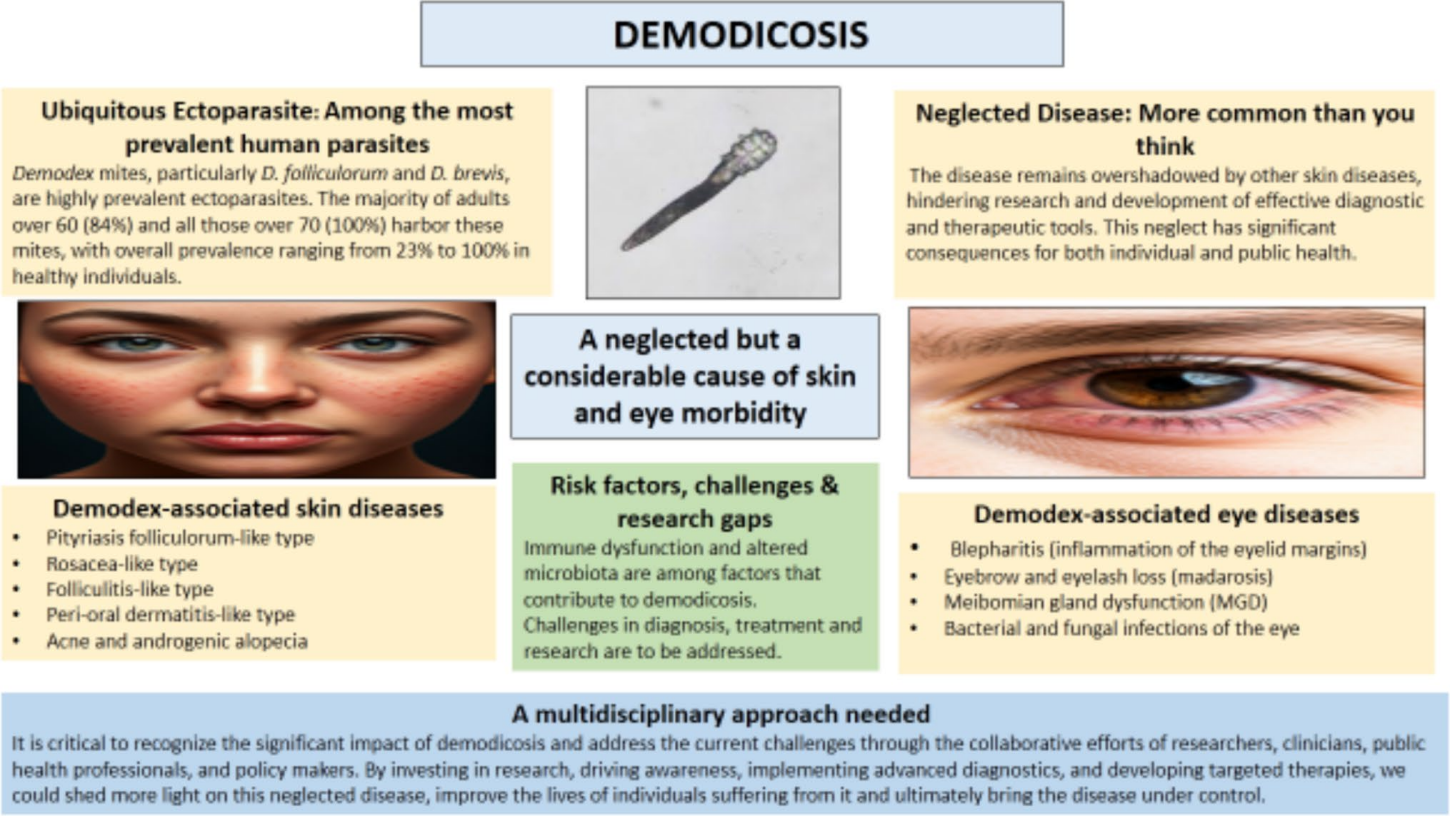

## Background

*BackgroundDemodex* mites, primarily *Demodex folliculorum* and *Demodex brevis*, are permanent residents of human hair follicles, particularly on the face. Several studies using molecular and other techniques have confirmed their near-universal presence in adults [1–3]. However, their commensalistic relationship with healthy individuals can shift, with *Demodex* proliferation leading to a spectrum of dermatological and ophthalmic conditions collectively termed demodicosis [4–6]. It has been recently suggested that *Demodex* is a stage in the transition from an obligatory parasite that harms the host to an obligatory symbiont [7].

Despite their prevalence, *Demodex* mites often remain asymptomatic in immunocompetent individuals. However, research suggests they may play a role in various eye and skin conditions in immunocompromised patients [4]. Uncontrolled mite populations can trigger an inflammatory response, manifesting as demodicosis. Notably, facial demodicosis frequently mimics other dermatological conditions like rosacea and folliculitis, leading to misdiagnosis [5, 6]. Similarly, *Demodex* infestation of the eyelashes can contribute to *Demodex* blepharitis, a chronic inflammatory eyelid condition that can lead to serious eye problems [8, 9].

Demodicosis is a prevalent but under-recognized disease. Studies report a prevalence of mite infestation in skin scrapings ranging from 10 to 55% [10–12]. Additionally, *Demodex* has been detected on the eyelashes of a significant proportion of healthy young adults [13–15]. Despite its high frequency, Demodicosis remains poorly recognized, which hinders its diagnosis, treatment, and research efforts. Delayed diagnosis and ineffective treatment strategies can lead to severe infections and disease spread, posing a burden on patients, communities, and healthcare systems.

To address the neglect of Demodicosis, a multi-pronged approach is necessary to raise public awareness, promote research, identify knowledge gaps, and develop better diagnostics and treatments. This review aims to contribute to this effort by highlighting the public health significance of demodicosis and potential strategies for reducing its burden. We advocate for more research on the understudied aspects of *Demodex* biology, pathogenesis, and clinical management. Our goal is to raise awareness of *Demodex* mites and their significant contribution to human diseases, as well as to elevate demodicosis on the global health agenda to improve public health outcomes and quality of life for affected individuals.

We conducted a literature review based on PubMed, Scopus, and Web of Science sources on January 15, 2023 and updated our search on January 25, 2025 to include all previously available literature up to this date. We set no limits on date of publication, nor on study design or language of publication. Search terms included “*Demodex*”, “mite”, “*folliculorum*”, “*brevis*”, “demodicosis”, “demodicidosis”, "neglect", “update”, “skin”, “eye”, “biology”, “epidemiology”, “pathogenicity”, “immune response”, “diagnosis”, “treatment”, “rosacea”, “acne”, “blepharities”, “seborrheic dermatitis”, “folliculitis”, “immune compromised host”, "opportunistic", “microbiota”, “microbiome”, “neglected disease”, "research", “challenges”, and combinations of these. One author (OME) carried out the initial search and screening of all papers, and the other (AAE) re-assessed the content of all papers. We found 249 articles relevant to the study aim. Preference was given to articles that provided a comprehensive overview of the biology, pathogenicity, diagnosis and treatment of *Demodex* mites and demodicosis, and the related discussions and challenges.

## Main text

### History and taxonomy of *Demodex* mites

*Demodex* mites were first observed by a German scientist, Jakob Henle, in 1841, but the discovery was not widely documented [16]. A year later, the German dermatologist, Gustav Simon, provided the first detailed description after examining material from acne lesions under a microscope. He named the worm-like creature *Acarus folliculorum* due to its movement and resemblance to an animal [17–20]. In 1843, a British scientist, Richard Owen, assigned the genus name *Demodex*, meaning "hair follicle boring worm" [20]. Two forms of *D. folliculorum* were later identified, with the larger one retaining the name and the smaller designated *Demodex brevis*. Initially considered subspecies, *Demodex brevis* was not recognized as a distinct species until 1963 [1, 19]. Further research explored the mites' anatomy, life cycle, presence of internal bacteria (endobacteria), lipase enzymes, and their ability to carry bacteria on their bodies [21–26].

*Demodex* is a saprophytic mite, a member of the Arachnida class, order Acarina, and family *Demodicidae* [20]; over 100 species have been recognized. Despite the fact that dermatologists, ophthalmologists, and veterinarians have been aware of *Demodex* mites for about 180 years, only two species—*D. folliculorum* (Simon, 1842) and *D. brevis* (Akbulatova, 1963)—have so far been linked to human demodicosis [1].

### Morphology

**Fig. 1:**
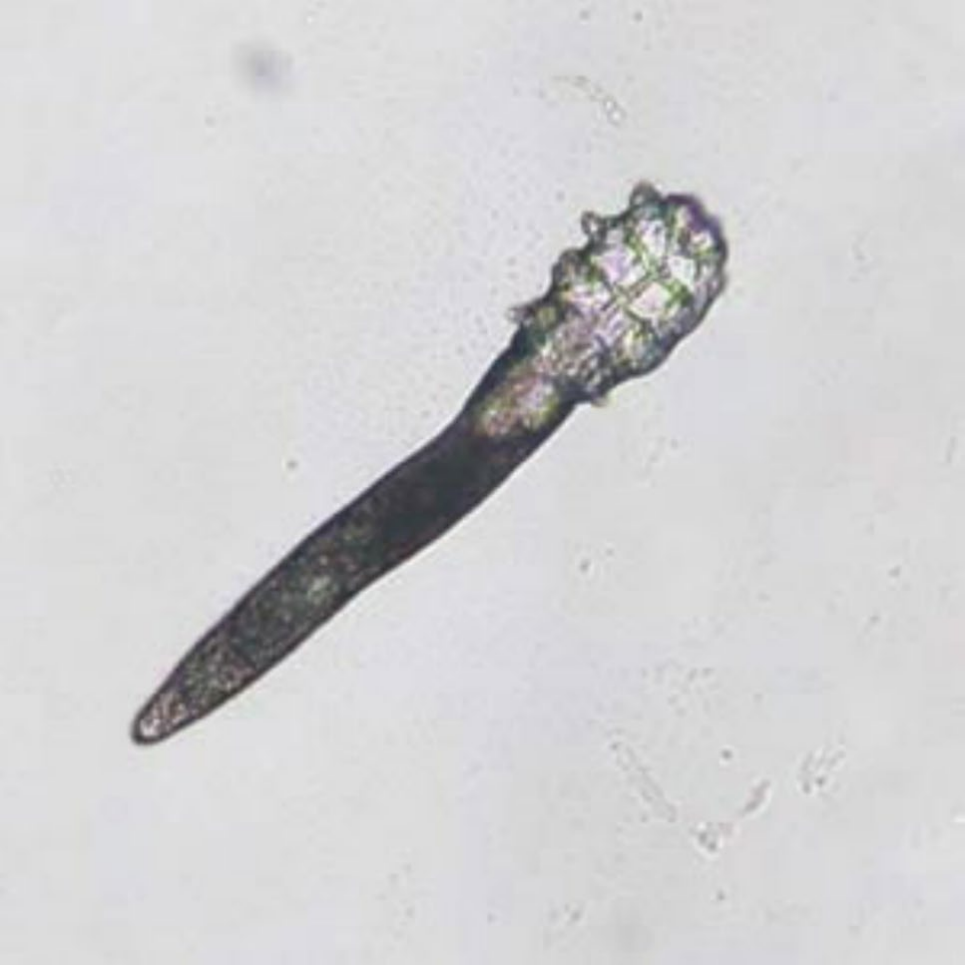
Adult *Demodex* mite. Image courtesy of DPDx, Centers for Disease Control and Prevention (https://www.cdc.gov/dpdx))

**Fig. 2:**
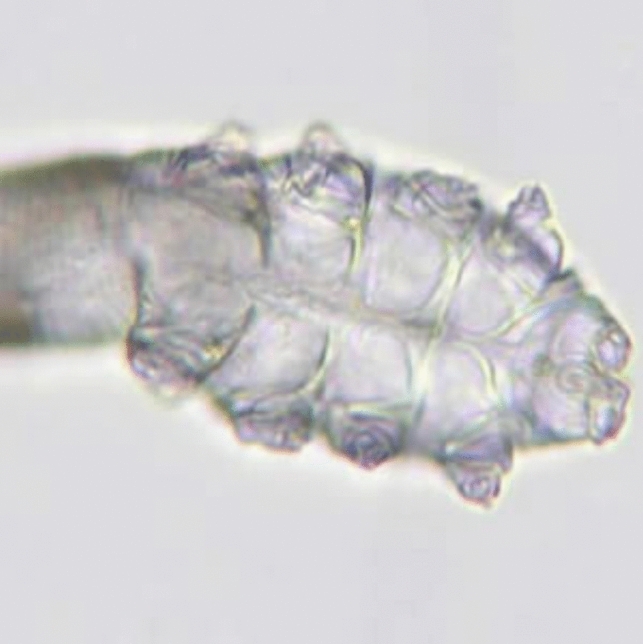
A close-up of the mouth parts (gnathosoma) and legs (podosoma) of the *Demodex* mite**.** Image courtesy of DPDx, Centers for Disease Control and Prevention (https://www.cdc.gov/dpdx)

Adult *D. brevis* mites measure 0.15–0.2 mm in length [27], while adult *D. folliculorum* mites are longer and thinner, measuring roughly 0.3–0.4 mm in length and 0.05 mm in width. Females are slightly shorter and rounder than males. They are undetectable to the human eye, yet their structure is readily discernible under a microscope [Figs. 1,2]. The mite’s elongated, semi-transparent body is made up of two segments that have fused together. The first body segment is connected to eight short, segmented legs. Scales on the body allow the mite to attach itself to a hair follicle, and its intricate pin-like mouth parts allow it to consume skin cells, oils (sebum), and other debris that build up in the follicles [27–29]. They have a rudimentary digestive system [17] and lipase enzymes are used in the digestive process [25].They have genitalia (vagina or penis, "aedaegus"). Recent investigations have suggested the presence of an anus in *Demodex* mites, refuting prior hypotheses that these ectoparasites lacked this essential anatomical structure [7]. In this recent research, high powered microscopy was used to discover this anatomical feature and has confirmed the existence of an anus in Demodex mites, visible through detailed microscopic observation of the mite's posterior region [7]. Contrary to the earlier belief that waste products accumulated within the mite's body throughout its lifespan, leading to potential inflammatory responses upon its demise, the anus is situated close to the end of the body on the ventral part of the abdomen that allow the mite to release waste products. The mite body has also a specialized protruding structure on the posterior end called the opithosomal organ. Although its precise purpose is not known, it is thought to be involved in reproduction by contributing to the production of pheromones or other mating-related signals. They may also secrete substances that interact with the host's skin environment, potentially influencing the host's immune response or the mite's own survival [2].

### Habitat

*Demodex* mites are obligate parasites with a specific preference for human skin [26]. They primarily target sebaceous glands and hair follicles, particularly on the face (eyebrows, lashes, nose, etc.) but can also be found on the scalp, ears, chest, and back [28, 29]. While *D. folliculorum* is more commonly found on the face, *D. brevis* is more likely to inhabit the neck and chest [30]. *D. folliculorum* typically resides near the base of the hair follicle, feeding on sebum and skin cells at densities below 5 mites/cm^2^ [28, 31, 32]. In contrast, *D. brevis* burrows deeper into sebaceous glands and ducts to access gland cells for sustenance [29]. Notably, *Demodex* infestation, particularly by *D. folliculorum*, can involve multiple mites residing within a single follicle, all facing the base (fundus) where sebum is most abundant [28, 33]. In severe cases of demodicosis, these mites may even penetrate the deeper dermal layer [2].

### Life cycle and biology

*Demodex* mites have a complex life cycle of approximately 14 days, although the exact details and optimal in vitro conditions for their growth remain elusive [23]. These obligate commensals reproduce internally, with both males and females possessing genital organs. Mating takes place within the hair follicle opening, followed by egg deposition inside the hair follicles or sebaceous glands. Six-legged larvae hatch within 3–4 days and mature into adults within a week [23]. The total lifespan of a *Demodex* mite is only a few weeks. They exhibit nocturnal activity, traveling at speeds of 8–16 mm/h, and retreat into follicles when exposed to bright light due to their photophobic nature [35].

Both temperature and the surrounding medium significantly impact *Demodex* viability in vitro [34]. These mites thrive in a temperature range of 16–22 °C, with activity levels decreasing at human body temperature (36–37 °C) [36]. Temperatures below 0 °C and exceeding 45 °C are detrimental, with 54 °C being lethal [34]. Interestingly, a combination of human serum and 1640/seroculture solution appears to be the most favorable laboratory medium for *Demodex* survival [36]. Recently, the study by Niu et al. (2024) [37] investigated the survival and morphological integrity of *Demodex folliculorum* under varying temperature and culture media conditions. Specimens were cultured at 16–22 °C and 4 °C in media including tea tree oil, phosphate-buffered saline, pure water, physiological saline, propidium iodide, liquid paraffin, glycerol, and a blank medium. Results demonstrated that 4 °C was optimal for in vitro survival with liquid paraffin yielding the longest survival time (12 days) and minimal morphological alterations in the mites.

### Prevalence, transmission and risk factors

*Demodex* mites, particularly *D. folliculorum* and *D. brevis*, are highly prevalent microscopic ectoparasites found on human skin worldwide [27, 39, 40]. Studies suggest that the majority of adults over 60 (84%) and all those over 70 (100%) harbor these mites, with overall prevalence ranging from 23 to 100% in healthy individuals [27, 38, 41]. The biological characteristics of *Demodex* mites significantly influence their transmission routes, primarily through direct skin contact. Additionally, sharing contaminated objects like makeup products used by multiple people over brief periods of time (ranging from a few hours to several days) may serve as a conduit for the spread of *Demodex* mites [47]. *Demodex* mites' obligate parasitic nature, limited off-host survival, and habitat specificity within the pilosebaceous unit restrict transmission primarily to direct or close contact. Their limited mobility and reproduction within the follicle further reinforce this. Even though vertical transmission from mother to infant can take place, the mites' biology renders indirect transmission by fomites or other indirect contact methods less likely because of the mites' limited environmental survival [1–3, 7]. While *Demodex* mites are widespread, most people are asymptomatic carriers and do not develop clinical signs of demodicosis. This suggests that the development of demodicosis is a multifactorial process influenced by both internal and external factors [42].

#### Risk factors

*Age—Demodex* prevalence is highest in young adults (20–30 years old) when sebum production peaks [43]. Mite numbers and the risk of demodicosis increase with age, while children under five rarely show signs of infestation [30, 44–46]. Newborns acquire *Demodex* through close contact after birth, but low sebum production in babies and young children limits extensive colonization [29]. Transmission from adults likely occurs in late childhood and early adulthood as sebaceous follicles mature [47]. Studies report varying prevalence in children and young adults, ranging from 12 to 70%, with some suggesting healthy children under 10 may not harbor *Demodex* [1, 44, 48, 49].

*Sex—*Males are more prone to *Demodex* infestation than females, with higher colonization rates (23% vs. 13%) and a greater abundance of *D. brevis* mites (23% vs. 9%), possibly due to men’s higher sebum production [28].

*Living conditions and animal contact—*While *Demodex* mites are highly host-specific, there are rare reports of dog mites (*Demodex canis*) potentially transferring to humans. However, definitive identification of *D. canis* requires further investigation as morphology alone can be unreliable, and *D. canis* may not establish itself on human skin even if transferred [50–52]. Studies suggest a possible link between increased *Demodex* prevalence in children from rural areas and their living conditions [49].

*Genetic predisposition—*Certain HLA (Human Leukocyte Antigen) types may be associated with resistance to demodicosis, suggesting a potential role for genetics in susceptibility [53, 54].

*Other risk factors—*Immunocompromised individuals, those with specific skin conditions like rosacea, blepharitis, and seborrheic dermatitis, users of topical or systemic corticosteroids, obese individuals, and people experiencing stress are all at increased risk for demodicosis [55–58]. The facial skin microenvironment, including factors like moisture, pH levels, sebum production, lipid composition, sebaceous gland function, vasodilation, and epidermal barrier integrity, can also influence *Demodex* mite proliferation [59, 60]. Certain medical conditions, such as diabetes mellitus and elevated blood sugar levels, may also favor *Demodex* growth [60].

In conclusion, *Demodex* mites are prevalent on human skin, but the development of demodicosis appears to involve a complex interplay between *Demodex* density, host factors like age, sex, and immune function, and the skin microenvironment. Understanding these risk factors is crucial for developing effective strategies for preventing and managing demodicosis.

### Pathogenesis and immune response

While generally harmless, an imbalance between *Demodex* mite density, immune function, and the skin microenvironment can trigger skin conditions [47].

*Increased mite density and immune suppression–* A high mite density (> 5 mites/cm^2^) or mite penetration into the deeper skin layers suggests potential for inflammation [28, 32, 61, 62]. Compromised immunity, either genetic or medication-induced, can worsen *Demodex* proliferation [57, 63–72]. Numerous immune-compromised human states have been shown to promote the population of *Demodex* mites and induce diseases, as explained in more detail below. Interestingly, *Demodex* themselves may contribute to local immunosuppression [54].

*Microenvironmental changes—*Facial skin pH, moisture levels, and lipid composition all influence *Demodex* populations. Changes favoring mite growth, like higher pH, have been linked to rosacea [73, 74]. *Demodex*-produced lipases further contribute to inflammation and hair follicle damage [25, 47, 75–80].

*Modified commensals and co-pathogens—*Disruptions in the skin microbiome, involving *Propionibacterium acnes* and *Malassezia* yeast, can alter the sebum composition of hair follicles and impact *Demodex* growth [74]. *Bacillus oleronius* bacteria are carried by *Demodex* mites in their abdominal cavity. Through neutrophil production and activation, this bacteria most likely acts as a co-pathogen and an exacerbating element in the development of the inflammatory process in rosacea, acne, and blepharitis [54, 81–83]. Additionally, *Streptococci* and *Staphylococci* on the surface of *Demodex spp.* are thought to be a possible cause of Meibomian gland dysfunction (MGD) and chalazion [84]. The second section of this review will give details about the interaction between skin microbiota and *Demodex* mites and their co-pathogens.

*Mechanical damage—Demodex* proliferation can mechanically obstruct follicles and glands, leading to tissue injury and barrier disruption [47]. Their presence can irritate the skin and trigger an immune response, contributing to disease [17, 78–80, 84, 85].

In conclusion, *Demodex* mites may contribute to skin disease through a combination of factors including mite density, immune function, skin microenvironment, interactions with commensals and co-pathogens, and mechanical damage. Understanding these interactions is key for developing effective treatments.

#### Immune response

*Demodex* mites trigger an immune response in humans through various mechanisms. Their proteins, waste products (detritus), and mechanical/chemical irritation of the skin all contribute to this response [86–89]. Toll-like receptors (TLRs) play a key role, with specific *Demodex* components activating TLR-2 in keratinocytes, leading to inflammation [90, 91]. Furthermore, *Demodex*-induced inflammation is mediated by increased pro-inflammatory cytokine production, particularly IL-17, from T lymphocytes [87–89]. In addition to the inflammatory response, *Demodex* may stimulate a humoral immune response involving immunoglobulin deposition (IgD) and cytokine secretion [62, 92]. Interestingly, *Demodex*-secreted bioactive molecules can modulate the immune reactivity of sebocytes, altering their TLR signaling pathway and cytokine production (IL-17, IL-8) [93]. These complex interactions between *Demodex* and the host immune system contribute to the development of demodicosis.

### *Demodex* and the skin microbiome

The skin microbiome, a diverse community of microorganisms residing on the skin surface, plays a critical role in maintaining skin health. These microbes, including bacteria, fungi, and mites like *Demodex*, contribute to antimicrobial defense, immune regulation, and overall skin homeostasis [3]. While the exact function of *Demodex* mites remains debated, some studies suggest they may aid defense against harmful bacteria [94].

The composition of the skin microbiome varies across different microenvironments. Dry, moist, and sebaceous areas harbor distinct bacterial communities. *Firmicutes*, *Actinobacteria*, and *Proteobacteria* dominate across these regions, with dry skin exhibiting the highest bacterial diversity [95, 96]. Specific bacterial genera like *Propionibacterium* favor sebum-rich areas, while *Staphylococcus* and *Corynebacterium* thrive in moist environments [97]. Disruptions in the microbiome composition are linked to various skin conditions [95–98]. Acne and rosacea are associated with sebaceous areas, while atopic dermatitis and body odor are prevalent in moist regions. Dry skin exacerbates psoriasis symptoms [95].

The relationship between *Demodex* mites and the skin microbiome is intricate. Arthropods are themselves home to symbiotic bacteria that can be useful or detrimental [99–102]. While some evidence suggests *Demodex* might contribute to a healthy microbiome, the exact mechanisms remain unclear. An imbalance in *Demodex* populations, particularly overgrowth, is suspected to play a role in certain skin conditions like rosacea [103]. Studies have identified correlations between *Demodex* abundance and specific bacterial populations. It has been suggested that a number of bacterial species are the endosymbiont of *Demodex spp*. mites. After finding acid-fast bacteria in the mites' digestive system, Spickett already proposed *Demodex spp.* as a leprosy vector in 1961 [104]. Rosacea patients often exhibit higher *Demodex* counts and distinct bacteria, *Bacillus oleronius*, compared to healthy individuals according to Lacey et al., 2007 [26], while increased *Propionibacterium acnes*, known to contribute to acne, coincides with *Demodex* presence [105]. It has been also reported that various strains of *Bacillus* species are related with *Demodex.* In 2016, Tatu et al. [106] discovered a strain of *Bacillus simplex* from *Demodex folliculorum*. Later in 2016, *Bacillus cereus* was also reported in patients who had rosaceiform face dermatitis brought on by topical steroids [107]. In 2017, Tatu et al. suggested *Bacillus pumilus* linked to *Demodex folliculorum* in rosacea lesional regions [108]. Clanner-Engelshofen et al. [109] in a more recent investigation, aimed to fill the knowledge gap regarding the endobacterial symbiont of *Demodex folliculorum* in a reproducible manner. They suggested that the vertically transmitted endosymbiont of *D. folliculorum* mites is C. *kroppenstedtii subsp. Demodicis*. Conversely, there is a negative correlation between *Demodex* and *Malassezia* yeasts, implicated in seborrheic dermatitis [110]. Interestingly, some research suggests a potential collaboration between *Staphylococcus* bacteria and *Demodex*. Bacterial antigens might suppress the host immune response, favoring the growth of both [111]. However, interactions between *Demodex* and other fungal elements, like *Malassezia*, have not been established.

#### *Demodex* coinfections

*Demodex* mites were believed to be potential vectors of infection transmission between people or from one area of the body to another because of their capacity to consume and spread a wide range of microorganisms found in their niche [112]. Recent research suggests a potential role for *Demodex* mites in the transmission of pathogens, while not definitively proven as vectors [84]. A growing body of research has shown associations with other pathogens and examined the co-infecting organisms of *Demodex* and the related symptoms. Examples include Liang et al.'s 2021 [113] research, which highlight the involvement of additional microorganisms, particularly bacteria, in ocular infestations and categorize them as co-pathogens of *Demodex spp*., such as *Novosphingobium*, *Acinetobacter calcoaceticus*, *AnoxyBacillus*, and *Pseudomonas* [113]. Furthermore, based on Hung et al.'s clinical observations from 2021 [114], it is found that patients with herpetic keratitis were also suffering from a concurrent *Demodex spp.* infestation. Blepharitis and rosacea have been linked to enhancement of the inflammatory response to the antigenic products and cathelicidin synthesis of *B. oleronius* bacteria of *Demodex spp.* [26, 115] in patients with papulopustular rosacea or ocular rosacea [116–118]. Furthermore, consideration is also given to the involvement of other bacteria, such as *Staphylococcus epidermidis* or *Staphylococcus aureus* with pathogenic potential, in the development of blepharitis, conjunctivitis, and pustular and ocular rosacea [87, 112, 117–119]. Additionally, co-infections with *Corynebacterium* and *Streptococcus* species implicated in blepharitis were noted [118, 119]. *Propionibacterium acnes* (*Cutibacterium acnes*) was found to cause both acne associated with *Demodex* and blepharitis [120]. Co-infection with *Microsporum canis* (fungus spores) and *Demodex* mite has also been found to cause dermatophytosis, pityriasis folliculorum, and other dermatological disorders [2, 112, 121].

It is important to note that the exact role of *Demodex* mites in pathogen transmission remains an area of active investigation. More research is necessary to establish a definitive link between these mites and the spread of infectious diseases.

### *Demodex* and immune suppression

*Demodex* mites, while generally harmless commensals on human skin, can flourish and contribute to skin disease under conditions of immunosuppression. This section explores the complex and sometimes contradictory relationship between *Demodex* and various immunosuppressive states [4].

#### *Demodex* and HIV/AIDS

Studies suggest a potential correlation between HIV infection and *Demodex* prevalence. Somsri and Wiwanitkit's research found a significant increase in *Demodex* mites on eyelashes of HIV patients with decreasing CD4 + cell counts [122]. However, larger studies are needed to confirm this association due to the limited research available and the heterogeneity of the immunosuppressed population [4, 123–125].

#### *Demodex* in organ transplant recipients

A few case reports link demodicosis to organ transplantation. Four cases of confirmed demodicosis were reported in kidney transplant recipients, suggesting a potential risk in this population [126]. Further investigation is warranted to determine the prevalence of demodicosis in this group.

#### Demodex and other immunosuppressive conditions

*Phototherapy*—Individuals undergoing phototherapy with ultraviolet (UV) light for skin conditions may experience an increased risk of demodicosis, possibly due to the immunosuppressive effect of UVA radiation compared to narrow-band UVB [127].

*Topical immunosuppressants*—Long-term or misuse of topical corticosteroids on the face has been linked to demodicosis [128–131]. Similarly, medications like tacrolimus and pimecrolimus, used to suppress T-cell activity in inflammatory skin conditions, may be associated with *Demodex* proliferation in some patients [132–134]. Four out of seven patients with tacrolimus-induced rosacea-like dermatitis and five out of six patients with steroid-induced rosacea-like dermatitis had higher densities of *Demodex* mites according to Teraki et al. (2012) [134]. While, Tatu (2016) [135] found that 32 of 40 patients with Topical Steroid Induced Facial Rosaceiform Dermatitis (TSIFRD) had dermoscopic features for *Demodex Folliculorum*.

*Discoid lupus erythematosus (DLE*)—While some studies suggest a link between DLE, a skin condition with an immunosuppressive component, and *Demodex* [136], others report a higher *Demodex* density in rosacea patients compared to DLE patients [137].

*Skin cancer*—*Demodex* infestation rates were found to be higher in patients with rosacea but did not differ significantly between controls and patients with basal cell carcinoma, squamous cell carcinoma, or DLE. Interestingly, patients with melanoma had lower *Demodex* densities, suggesting a potential dissociation between *Demodex* and immunosuppression associated with melanoma treatment [138].

*Other medical conditions*—Studies have explored potential links between *Demodex* and other conditions like diabetes, allergic rhinitis, and heart failure. While some studies suggest an association, the evidence is limited and requires further investigation [139, 140].

In conclusion, the relationship between *Demodex* and immunosuppression appears multifaceted. While some studies suggest a connection, others do not. The type, severity, and duration of immunosuppression may influence this relationship. Further research with larger and more homogenous patient populations is needed to establish if there is a definite link between *Demodex* and various immunosuppressive conditions.

## Clinical presentations

### *Demodex* and skin diseases

**Fig. 3:**
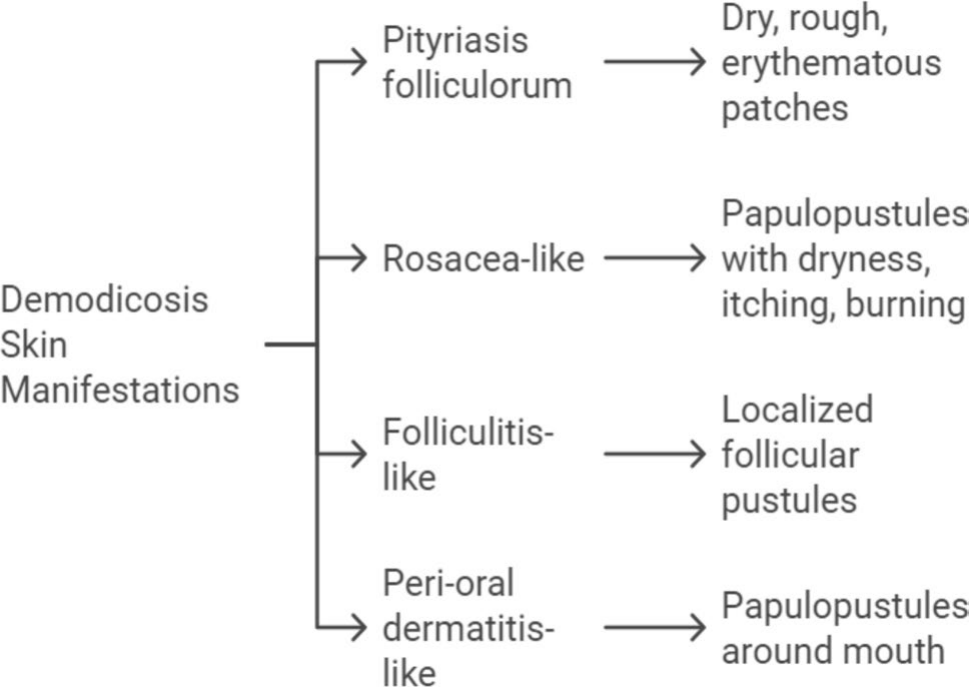
Skin conditions linked to *Demodex* mites

Demodicosis, encompassing various skin conditions caused by *Demodex* mites, presents with a spectrum of clinical features and severity as shown in Fig. 3 [40, 141]. These features depend on mite density, the skin microenvironment, and the host's immune response. Manifestations can range from non-specific dryness and sensitivity to papules, nodules, and even granulomas [47]. There are two main clinical types: (1) Primary demodicosis involves an unexplained increase in mite colonization leading to inflammatory skin lesions that persist without treatment [40]. This type encompasses conditions like pityriasis folliculorum, nodulocystic demodicosis, and blepharitis [39, 40]; and (2) Secondary demodicosis refers to the presence of *Demodex* mites in individuals with pre-existing skin diseases or systemic conditions, often seen in immunosuppressed patients who may experience a wider range of symptoms than immunocompetent individuals [39, 40].

Clinically, demodicosis manifests in four main categories:

- Pityriasis folliculorum type: presents with dry, rough, erythematous patches on the face due to increased follicular scaling [142, 143].
- Rosacea-like type: characterized by papulopustules on the face, with or without rosacea, accompanied by dryness, itching, and burning [144].
- Folliculitis-like type: localized follicular pustules resembling folliculitis or acne [85, 145].
- Peri-oral dermatitis-like type: papulopustules around the mouth mimicking peri-oral dermatitis [146].

Pityriasis folliculorum is the most common presentation, followed by rosacea-like and peri-oral dermatitis-like types [146]. Demodicosis can also manifest as unexplained eczema, scalp issues (pruritus, dandruff, folliculitis), seborrheic dermatitis-like lesions, *Demodex* abscesses, granulomatous lesions, and even involve the ears and vulva [142, 147–154]. More information on the *Demodex*-related skin and ocular diseases is provided below.

#### Rosacea and *Demodex*

Rosacea, a chronic inflammatory facial skin condition characterized by redness, pustules, and dilated blood vessels, has an unclear etiology [155]. Multiple factors are suspected, including abnormal blood flow in the face, dysregulated inflammatory responses, and the overgrowth of resident skin microorganisms [91]. *Demodex* mites have been considered the cause of facial rosacea since the 1930s due to their potential to activate these pathways and their increased abundance on rosacea patients' skin [84, 156]. According to certain theories, the pathogenic potential is correlated with an increase in mite density (above 5 mites/cm^2^). That is why there is a seasonal worsening of rosacea during the warmer spring and summer months when mite populations might flourish [84].

Topical antibiotics (metronidazole 0.75%) were shown to be more effective in treating rosacea than topical anti-*Demodex* cream (permethrin 5%), despite the fact that the latter lowered *Demodex* levels [157]. This suggests that bacterial involvement may also play a role in rosacea pathogenesis. The combination of *Demodex* mites and the *Bacillus oleronius* bacteria, they are connected with, may synergistically trigger inflammatory pathways in rosacea patients [158, 159]. Additionally, *Staphylococcus epidermidis*, another bacterium potentially associated with *Demodex* mites, has been found in follicular biopsies from rosacea patients [26]. These findings suggest a multifaceted interplay between *Demodex* mites, bacteria, and the host immune response in rosacea development. It is also important to recognize that while *Demodex* mites may play a role in the development of rosacea, rosacea itself may also be a sign of demodicosis. This viewpoint draws attention to the complex interactions between these factors and stresses how important it is to comprehend how they are related.

#### Acne and *Demodex*

The potential link between *Demodex* mite infestation and acne vulgaris is a topic of ongoing debate in dermatology. Several studies support this association [160–162]. A meta-analysis by Zhao et al. evaluated 63 articles from various nations on 42,130 individuals and found a significantly higher *Demodex* infestation rate in acne patients compared to controls [161]. The overall Demodex mite infestation rate was 54.9% in acne patients, which was 31.5% higher than in the control group, suggesting a positive correlation between *Demodex* and acne development. However, other studies, such as those by Okyay et al. (2006), and Paichitrojjana et al. (2024) have reported no significant association between *Demodex* density and acne prevalence [163, 164].

Proponents of the *Demodex*-acne link point to several observations. First, individuals with oily or combination skin, more prone to acne, also exhibit higher *Demodex* infestation rates compared to those with normal skin [160]. Second, factors like hyperandrogenism, obesity, and insulin resistance, which contribute to acne development, may also increase susceptibility to *D. folliculorum* infestation [165–170]. Additionally, Yarim et al. demonstrated elevated insulin-like growth factor 2 (IGF-2) levels in dogs with demodicosis, mirroring the increased levels observed in acne patients [171].

However, there are several counterarguments to this proposed relationship. Even in people without acne, *Demodex* mites are very common, which calls into question their causal significance [84]. Establishing a clear cause-and-effect relationship between *Demodex* mites and acne is difficult. While some studies have shown a higher prevalence of mites in individuals with acne, correlation does not necessarily imply causation. Acne is multifactorial, encompassing hormones, environmental factors, and genetics, making it challenging to establish causation [172]. Additionally, the specific mechanisms by which *Demodex* mites might contribute to acne are not fully understood. There is yet no proof that *Demodex* causes acne through the suggested pathways of inflammation or disturbance of the microbiota, and more research is needed to confirm these potential mechanisms. Lastly, conflicting study results call for more investigation to determine the exact connection between *Demodex* mites and the onset of acne.

The clinical similarity between *Demodex* folliculitis and acne vulgaris, with papules, pustules, and nodules, further complicates diagnosis. Dermatologists may misclassify *Demodex* folliculitis as acne due to the absence of comedones, a hallmark of acne but not *Demodex* folliculitis [85].

While the causal relationship between *Demodex* and acne remains unclear, considering *Demodex* as a potential contributing factor in some acne cases may be a prudent approach. Some advocate for *Demodex* testing and treatment with acaricides in acne patients who fail to respond to conventional therapies [85].

In conclusion, despite some data pointing to a potential connection between Demodex mites and acne, there are a number of considerations that refute this notion. Uncertainty around this topic is exacerbated by the mites' broad presence, the challenge of proving causation, the ambiguous mechanisms of action, and the scant and contradictory research. More research is needed to completely understand the involvement of *Demodex* mites in the development of acne.

#### Pityriasis folliculorum and *Demodex*

*Pityriasis folliculorum*, characterized by facial redness, dryness, scaling, and itching is an early manifestation of *Demodex* overgrowth (mite density around 60/cm^2^) distinct from seborrheic dermatitis and rosacea. Later presentations may include papulopustular, acneiform, or maculopapular rash without comedones or telangiectasia, potentially associated with even higher mite densities [173–176].

#### Androgenetic alopecia and *Demodex*

*Demodex* mites have been linked to androgenetic alopecia. While the exact relationship remains unclear, *Demodex*-secreted lipases may trigger inflammation around sebaceous glands. This inflammation, coupled with the follicular infiltration of immune cells, can lead to fibrosis and hair follicle loss [177, 178]. Additionally, the inflammatory response might alter local hormone metabolism, promoting sebum production and creating a more favorable environment for *Demodex* growth. Ultimately, chronic *Demodex* infestation may contribute to hair cycle disruption and hair loss [177].

#### Basal cell carcinoma (BCC) and *Demodex*

There have occasionally even been suggestions linking *Demodex* mites to BCC. Some studies have suggested a possible link between high *Demodex* mite populations and BCC [84, 179]. However, these findings are not conclusive, and further research is needed to establish a definitive causal relationship. In addition to UV radiation being the primary cause of BCC, attention is also paid to local variables such as inflammation, irritation, or chronic damage. Chronic inflammation caused by *Demodex* in areas like the nose and eye sockets, which are commonly affected by BCC, raises the possibility of *Demodex* involvement in BCC. Consequently, the significance of demodicosis as one of the mechanisms driving carcinogenesis in BCC of the eyelids in predisposed individuals is also stressed [179] because of the irritating/traumatic effect that induces persistent inflammation. Moreover, Sun et al., 2005 [180] analysis demonstrated that a high infestation rate among the malignancies under study was a defining characteristic of BCC cases. According to a recent study in 2023 [181], the inflammatory response triggered by *Demodex* mites in rosacea may help create an atmosphere that is favorable for the development of BCC. 46.4% individuals with BCC who had concurrently diagnosed rosacea were identified. Finally, it is critical to consider the distinction between causation and correlation. Although research may indicate a link between *Demodex* mites and BCC, this does not imply that the mites are the direct cause of BCC, which has a complex etiology. The potential contribution of *Demodex* mites is likely to be one of many contributing factors. According to a systematic review in 2023, the role of *Demodex* mites in the development of skin cancer remains a subject of ongoing research [84].

#### Miscellaneous *Demodex*-associated skin conditions

*Demodex* mites have been implicated in various other skin conditions, including perioral dermatitis, acariciariform dermatitis, Grover's disease, eosinophilic folliculitis, and facial/scalp eruptions [182]. The inflammatory response to *Demodex*, along with other microorganisms like bacteria, yeasts, and fungi in hair follicles, might contribute to dissecting cellulitis of the scalp [84, 183, 184]. Additionally, some authors have proposed *Demodex* as a potential trigger for Lupus Miliaris Disseminatus Faciei (LMDF), but this requires further investigation [185]. Excessive makeup use has also been linked to *Demodex*-related issues like scabies-like sores, scalp eruptions, and dandruff [84]. Additionally, dermal nevi have shown increased *Demodex* colonization, suggesting that the mite prefers melanin pigment [182].

Other recent reports have shown that* Demodex* mites are associated with the following presentations:

##### Pityriasis folliculorum of the back thoracic area

A case report describes a patient presenting with multiple filiform spicules, white-yellowish keratotic follicular plugs, and small angiomas on the posterior thorax [186]. Notably, numerous non-hair-bearing follicular orifices exhibited minute, hyperkeratotic spicules, and the skin exhibited a sandpaper-like texture on palpation. Dermoscopic examination revealed filiform threads and semi-round white plugs within follicular openings. Parasitological assessment confirmed the presence of *Demodex folliculorum*. Based on these findings, a diagnosis of thoracic pityriasis folliculorum caused by *D. folliculorum* was established.

##### Pigmented demodicidosis

A recent report describes a distinct clinicopathological and dermoscopic presentation of facial hyperpigmentation associated with *Demodex* mites [187]. Nineteen patients presented with dusky brown-gray pigmentation, either localized or diffuse, often accompanied by erythema and skin roughness. The authors propose the term "pigmented demodicidosis" and advocate for its inclusion in the differential diagnosis of facial hyperpigmentation.

##### Relationship between *Demodex* and SARS-CoV-2

Some researchers hypothesize that arthropods inhabiting human skin, such as *Demodex* mites, may play a role in the transmission of viruses, including SARS-CoV-2 [188]. This hypothesis suggests a potential interaction between the chitinous exoskeleton of the mite and the lipid envelope of the virus, mediated by molecular attraction forces. If confirmed, this could establish arthropods like *Demodex* as an overlooked cofactor in viral infections, with significant implications for disease prevention and treatment strategies.

It is important to note that the strength of the evidence for *Demodex* involvement in these conditions varies: while some associations show promise, others require further research to establish a definite link.

### *Demodex* and eye diseases

**Fig. 4:**
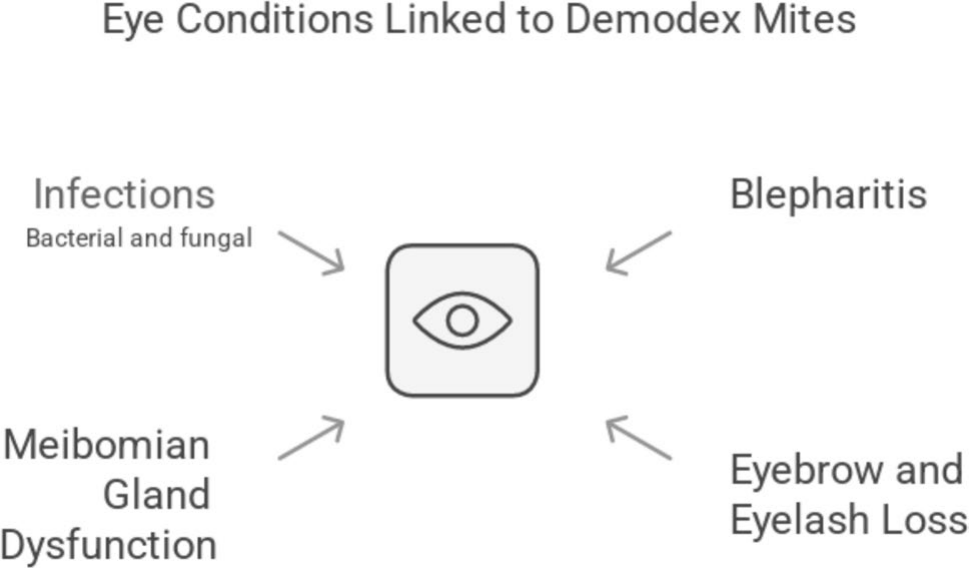
Eye conditions linked to *Demodex* mites

*Demodex* mites, commonly found on human skin, can also colonize the eyelids, potentially contributing to various eye diseases as shown in Fig. 4. Patients with demodicosis often have higher levels of eyelash mites compared to healthy individuals, highlighting the potential for mite migration from facial skin to the eyelids [13–70% of blepharitis cases worldwide have been linked to mite infection] [39]. Therefore, eyelash mites should be examined in all demodicosis patients to prevent potential eye complications [73].

*Demodex* mites have been implicated in several eye conditions, including:

- *Blepharitis (inflammation of the eyelid margins)*: Both anterior (characterized by a crusty eyelash base) and posterior (marked by symmetrical eyelid bumps) blepharitis can be associated with *Demodex* infestation by either *D. folliculorum* or *D. brevis* mites [2, 87, 118, 189–195]. Unilateral blepharitis with fine follicular scaling was reported in a patient following an 8-week course of topical steroid use. *Demodex folliculorum* infestation was confirmed [196]. Untreated blepharitis can lead to serious complications like corneal ulcers and permanent eyelid changes [84].
- *Eyebrow and eyelash loss (madarosis)*: *Demodex* mites can contribute to eyebrow and eyelash loss [189].
- *Meibomian gland dysfunction (MGD)*: Blockage of meibomian gland openings by *Demodex* mites, along with bacterial/fungal infections, can lead to MGD, a condition affecting the quality of tear film [84].
- *Bacterial and fungal infections of the eyes*: *Demodex* infestation may create a favorable environment for bacterial and fungal eye infections (including *Staphylococcus aureus*, *Corynebacterium spp.*, and *Bacillus oleronius*) [84].

The mechanisms by which *Demodex* mites contribute to eye disease are multifaceted:

- *Inflammation*: *Demodex* mites can trigger inflammation through the release of cytokines and allergic reactions to their chitinous exoskeletons [84].
- *Bacterial/fungal co-infections*: *Demodex* infestation might create a niche for bacterial and fungal pathogens to thrive, further worsening eye health [120, 197].
- *Obstruction of meibomian glands*: Both *Demodex* species can physically block the openings of meibomian glands, hindering tear secretion [84].
- *Corneal damage*: Chronic irritation from *Demodex*-related blepharitis can damage the cornea [193].

*Demodex* mites have also been linked to recurring chalazia and styes, dry eye syndrome, and even endophthalmitis [9, 86, 170]. Some studies suggest a potential role for *Demodex* mites in the development of basal cell carcinoma of the eyelids [86, 179], however, further research is needed to solidify this association.

In conclusion, *Demodex* mites are emerging as significant contributors to various eye diseases. Early diagnosis and management of *Demodex* infestation are crucial to prevent vision-threatening complications.

### Public health impact of Demodex mite infections

Neglecting *Demodex* mites may have significant direct and indirect consequences on individual and public health:

- Direct impact on individuals:

*Demodex* mites can cause various skin and eye conditions. These conditions can lead to discomfort and disfigurement. The severe itching, redness, and visible skin lesions can have a major negative effect on a person's quality of life by resulting in emotional discomfort and social anxiety. Vision problems, blurred vision, and even corneal ulcers can be consequences of *Demodex* infection if left untreated. Increased infection risk is high because *Demodex*-induced skin and eye inflammation can weaken the host's protective barriers, raising the possibility of subsequent bacterial or fungal infections [40, 47, 122, 141, 190].

- Indirect public health implications:

Public health consequences of *Demodex* infections include economic burden: The treatment of *Demodex*-related diseases can be costly, placing a financial burden on individuals and healthcare systems. Economic losses are also influenced by lost productivity as a result of missed work or school days. Social stigma: Rosacea and other skin disorders can be stigmatizing and cause social isolation and anxiety. Potential for transmission: Although not very contagious, close contact can spread *Demodex* mites, particularly in households, nursing homes, and child care centers. Transmission may result in more serious infections in people who are immunocompromised or have deficient skin barriers [2, 4, 40, 47, 49, 84].

Overall, even though the direct public health effects of *Demodex* mite infections may not seem as great as those of highly contagious diseases, neglecting demodicosis can have significant consequences for individuals and public health, including reduced quality of life, increased healthcare expenses, and social and financial burden. Therefore, it is important to increase public awareness about *Demodex* mites and their potential impact on public health in order to promote appropriate diagnosis and treatment.

### *Demodex* diagnosis: evolving techniques and challenges

*Demodex* mites, despite their association with various skin and eye conditions, are often underdiagnosed [161]. This stems from the lack of a standardized diagnostic method. Demodicosis is thought to be caused by a high density of *Demodex* mites. Thus, the technique for calculating the mite density per square centimeter plays a key role in the diagnosis of demodicosis.

Several techniques are employed to collect mite samples for analysis:

- *Cellophane tape method*: A simple approach involving pressing cellophane tape onto the skin and examining it under a microscope for mites [198].
- *Squeezing method*: Directly expressing follicular contents by squeezing a defined skin area with a comedone extractor [23].
- *Skin scrapings*: Scraping the skin surface with a scalpel to collect sample material [3].

*Standardized Skin Surface Biopsy (SSSB)*: This non-invasive method, considered the gold standard for comparing mite densities between patients and healthy controls, involves applying cyanoacrylate glue to a slide, pressing it onto the skin, and examining the collected sample under a microscope after gentle removal [32, 33, 199]. A 1 cm^2^ area is typically marked on the slide for accurate density calculation.

*Direct Microscopic Examination (DME)*: This technique involves squeezing a defined skin area with a comedone extractor and examining the expressed material under a microscope with potassium hydroxide [146].

Both SSSB and DME samples are examined under a microscope to count *Demodex* mites. A density exceeding 5 mites/cm^2^ is generally considered indicative of *Demodex* [32]. While SSSB offers higher sensitivity than DME in measuring mite density, the validity of the 5 mites/cm^2^ threshold as a diagnostic cut-off is debated due to limited supporting data [40, 200].

*Skin Punch Biopsy*: Although less common due to its invasive nature, this method can detect mites deeper within the follicle compared to SSSB. This explains the higher *Demodex* densities observed in skin biopsies compared to SSSB, suggesting deeper follicular localization for most *D. folliculorum* mites [201].

*Superficial Needle Scraping (SNS)*: This technique, proposed by Huang, involves scraping pustules with a needle tip for *Demodex* examination. A density of 3 or more mites per 5 pustules is considered diagnostic for demodicosis in papulopustular rosacea (PPR) [202]. The author suggests combining SNS with Gram staining and SSSB for a comprehensive evaluation of *Demodex*-linked facial papulopustular eruptions [47].

*Dermoscopy, Reflectance Confocal Microscopy (RCM), and Confocal Laser Scanning Microscopy (CLSM)*: These non-invasive imaging techniques offer promising advancements in *Demodex* diagnosis and treatment monitoring [47, 203–205]. They provide high-resolution (RCM and CLSM) or low-resolution (dermoscopy) in situ visualization of skin features. Dermoscopy can reveal *Demodex*-related signs like "*Demodex* tails" (gelatinous filaments emerging from follicles) and "*Demodex* follicular openings" (dilated follicles with erythematous halos) [206]. While these techniques can identify and measure mites per follicle, limited visibility precludes biological examination. Nevertheless, they represent valuable diagnostic tools despite their cost.

For diagnosing *Demodex* involvement in the eyelids, microscopic examination of a few epilated eyelashes mounted on a slide with Hoyer's liquid is employed [207, 208].

#### Diagnostic challenges

Diagnosing *Demodex* remains a challenge due to the lack of a standardized approach and is complicated by factors beyond *Demodex* density alone. Demodicosis is a complex disease; alterations in the skin microenvironment and host immunity are equally important as the aberrant proliferation of *Demodex* mites, which is the primary focus of different diagnostic techniques. Although this is not always the case, patients with demodicosis tend to experience more severe clinical symptoms when the density of *Demodex* mites is high [146]. On the other hand, people with normal skin can have a high density of *Demodex* mites without exhibiting any clinical signs of the disease [44]. According to research study, not all patients showed significant clinical improvement from demodicosis and a density of *D. folliculorum* at less than 5 D/cm^2^ following treatment [142]. The patients' host immunity response was the main cause of all of these events. Erbagci and Ozgoztasi proposed that a specific density might not be a proper indicator in the diagnosis of rosacea; nonetheless, in addition to other stimulating variables, a high concentration of *D. folliculorum* may play a significant part in the pathogenesis of rosacea [62]. Hence, it is not appropriate to base decisions on the precise quantity of *Demodex* mites present. This is because, although the majority of *Demodex* mites are found deep within hair follicles, they can exit the follicles and travel on the skin's surface [27, 201]. SSSB is only able to identify *Demodex* mites on the skin's surface. This could result in erroneous negative findings when measuring *Demodex* mites [61]. Numerous investigations have demonstrated that there were variations in the quantity of *Demodex* mites discovered using various testing techniques [146, 200]. Therefore, the diagnostic criteria should include relevant link between suspected clinical skin lesions and the presence of aberrant *Demodex* mite proliferation, as well as clinical cure and normalization of *Demodex* mite density following acaricidal treatment [47].

In conclusion, diagnosing *Demodex* remains a challenge due to the lack of a standardized approach and other factors. However, various techniques, including SSSB, DME, skin punch biopsy, SNS, and non-invasive imaging modalities, offer a growing toolkit for accurate *Demodex* diagnosis and effective treatment monitoring.

### *Demodex* treatment: balancing efficacy and challenges

*Demodex* mites pose a therapeutic challenge. While various treatments exist, including oral and topical medications, none offers a perfect solution [209]. Treatment goals focus on inhibiting mite reproduction, eliminating existing mites, and preventing re-infestation [192].

#### Antiparasitic and antibacterial therapies

- *Oral medications*: The combination of antibiotics like tetracycline/doxycycline with acaricides like ivermectin/permethrin supports the theory of endosymbiotic bacteria contributing to demodicosis [209, 210]. Metronidazole, an antibiotic employed in the treatment of various anaerobic bacterial and parasitic infections, is also utilized in the management of *Demodex* infestations. Its mechanism of action involves the disruption of the pathogen's DNA structure [84]. Studies show promise for oral metronidazole, and ivermectin, with a 71.6% complete remission rate in rosacea and blepharitis patients [210]. Ivermectin alone also yielded significant improvement in 45% of patients.
- *Topical medications*: Topical ivermectin, which combats *Demodex* mites externally and internally, offers an alternative to oral medications, exhibiting anti-inflammatory, antibacterial, and antiparasitic properties [211]. Studies show remarkable clinical improvement and mite eradication with 1% topical ivermectin after 16 weeks [212], while 10% benzyl benzoate showed little effect [213]. Topical metronidazole (2%) is recommended for demodicosis blepharitis and conjunctivitis [31]. However, caution is advised with topical treatments due to potential skin sensitivity and irritation reported with several options [214, 215]. Oral medications are often reserved for severe cases or immunocompromised individuals.

#### Alternative and supportive measures

- *Medicinal oils*: Tea tree oil, with its diverse properties (antiseptic, antibacterial for aerobic and anaerobic bacteria, antifungal, antiviral, antiparasitic, anti-inflammatory, regenerative), has gained traction for its potential to reduce mite numbers, alleviate symptoms, and modulate the immune system [216–220]. Additionally, it possesses regenerative and anti-inflammatory qualities, speeds up wound healing and epidermal renewal, and may even be able to cure ulcers. Studies have shown a decrease in *Demodex* mites on eyelids with tea tree oil ointment, suggesting its use as an adjunctive therapy [221]. Other medicinal oils such as camphor oil, bergamot oil, peppermint oil, salvia oil, black seed oil (Nigella sativa oil), castor oil, as well as sulfur ointment, yellow or white mercury ointment, and choline esterase inhibitors have also been used in the treatment of skin demodicosis [39, 216]. These medicinal oils demonstrate potential efficacy against *Demodex* mites through various mechanisms of action [84]. Salvia oil has been shown to decrease mite vitality. Peppermint oil exhibits antiseptic properties. Castor oil possesses antibacterial, anti-inflammatory, analgesic, antioxidant, and wound-healing properties, in addition to promoting eyelash growth and inhibiting hair loss. Black seed oil accelerates wound healing and moisturization, strengthens and softens the skin, and improves skin tone. Finally, bergamot oil exhibits antiseptic, cooling, and soothing properties, alongside antibacterial, antiviral, antifungal, and antiparasitic actions.
- *Physical therapies*: Infrared radiation therapy and customized heating glasses are additional treatment modalities employed for demodicosis [222]. Sun exposure and washing the face with warm water have also been shown to be beneficial [192]. Microblepharoexfoliation, utilizing a high-speed rotary sponge with lid cleaner effectively removes biofilm and mite eggs from the eyelid margins [192]. Intense Pulsed Light (IPL) therapy has demonstrated potential efficacy in treating demodicosis, including in cases of rosacea and ocular infestations. Although the precise mechanism remains unclear, it is hypothesized that IPL may eradicate *Demodex* mites by exposing them to elevated temperatures generated during treatment [182, 223].
- *Herbal remedies*: Herbal and plant extracts with antiparasitic properties, like those from celandine, calamus, or mugwort, can be used for eyelid hygiene [222].
- New therapeutic agents:

Emerging therapeutic agents for *Demodex* infestation include:

- XDEMVY™ (lotilaner ophthalmic solution) 0.25% is the first FDA-approved treatment for *Demodex* blepharitis. XDEMVY is a prescription eye drop that effectively eradicates *Demodex* mites by targeting their nervous system and selectively inhibiting the GABA-Cl (Gamma-Aminobutyric Acid- activated Chloride) channels. Clinical trials demonstrated its efficacy and safety, with minimal side effects primarily consisting of mild eye irritation [224].

- Manuka Honey: Containing cyclodextrin-complexed methylglyoxal (MGO), Manuka honey exhibits anti-*Demodex* activity comparable to tea tree oil while demonstrating excellent tolerability [225].

- Hypochlorous Acid: This compound possesses antimicrobial properties [226].

- Okra-derived Polysaccharide: This natural substance also shows potential antimicrobial activity [227].

##### Treatment challenges

- *Limited evidence base*: Standardized treatment protocols and long-term drug efficacy for demodicosis remain elusive [214]. Treatment selection is individualized and can be a complex, multi-month process, often relying on case reports with limited robust evidence [40].
- *Confounding factors*: Distinguishing between inflammatory diseases (e.g. rosacea with/without secondary demodicosis) and primary demodicosis poses a diagnostic challenge, further complicating treatment strategies.
- *Dual-action medications*: Many therapeutic agents possess both anti-inflammatory and antimicrobial properties, making it difficult to isolate their specific effects on *Demodex* mites.
- *Addressing co-infections*: Eradicating *Demodex* mites might not fully resolve the condition, as secondary bacterial infections and inflammation can persist [116]. Extended-spectrum antibiotics might be necessary to address co-infections [8]. Combining antibacterial and anti-degenerative medications has shown promise in improving patient outcomes [228].

In conclusion, *Demodex* treatment requires a multifaceted approach, considering the limitations of current therapies and the potential for co-existing conditions. Further research is necessary to establish standardized treatment protocols and identify more effective medications for long-term *Demodex* management.

### *Demodex*: questions and research gaps

*Demodex* mites, while commonly found on human skin, remain shrouded in mystery. Our understanding of their role in various skin diseases is far from complete. This highlights several critical areas requiring further investigation:

*Demodex and disease causation:*

- Are *Demodex* mites the primary culprits behind skin diseases, or are they merely bystanders? Conflicting research findings need larger, controlled studies to answer this basic question. A reliable diagnostic test like SSSB, accurately measuring mite density, could strengthen the link between *Demodex* infestation and conditions like rosacea and acne [47, 161].

*Clinical manifestations and disease definition:*

- The precise definition and classification of demodicosis are still debated [40, 141]. Beyond the typical symptoms, a wide range of clinical presentations have been documented, including pityriasis folliculorum, rosacea-like features, and perioral dermatitis [129, 147]. Even non-specific facial symptoms like unexplained itching and papulopustular lesions can be associated with *Demodex* proliferation [142, 148]. More research is needed to solidify the connection between *Demodex* and these diverse clinical manifestations, ultimately refining the definition of demodicosis and clarifying the mites' role.

*Long-term consequences and mite biology:*

- The long-term implications of *Demodex* infestation are poorly understood. Some studies suggest a potential association between chronic infestation and an increased risk of basal cell carcinoma. Further investigation is necessary to validate these findings [84].
- Detailed knowledge regarding the *Demodex* life cycle and behavior is lacking. Understanding factors like the male-to-female mite ratio within hair follicles, their day-night activity patterns, and their presence within vs. between follicles is crucial for a more comprehensive picture.

*Demodex, immunity, and pathogenesis:*

- Our understanding of the immune response and pathogenesis in demodicosis is limited. The precise interaction between how the human immune system and *Demodex* mites lead to disease development is unclear. We need to explore immune responses, particularly comparing the innate immunity of healthy versus diseased individuals. Other areas requiring investigation include the relationship between *D. folliculorum* and *D. brevis* in various body regions, mite virulence factors, and the link between mite density and clinical disease activity [84].

*Risk factors and host susceptibility:*

- The factors contributing to *Demodex* proliferation, such as the age-related increase (nearly 100% prevalence in older adults), need further exploration. Studies on the role of immune system status and genetic predisposition could identify individuals more susceptible to *Demodex* infection, allowing for targeted prevention strategies.
- The link between immunosuppression and *Demodex* infection requires further study. Currently, there is only limited data from case reports and small studies. Larger trials involving immunocompromised patients are needed to confirm potential correlations.

*Demodex and the skin microbiota:*

1. The intricate relationship between *Demodex* and the skin microbiota needs further elucidation. Understanding how *Demodex* interacts with the skin's bacterial communities is essential. Advances in microbiome research could shed light on *Demodex*'s role as a disease agent or a carrier of other bacteria. Additional studies are needed to identify pathogens associated with *Demodex*-related skin disorders and compare the microbial composition in patients with and without *Demodex* infestation [2, 229].

*Demodex culture and genetic research:*

- Developing a reliable *Demodex* culture system is an urgent need. Currently, the mites die rapidly and cannot be maintained in vitro. Since human tissue is the only known source of *Demodex*, a culture model would revolutionize research opportunities, eliminating the dependency on live animals [230]. In vitro cultures would facilitate research on the *Demodex* life cycle, biology, physiology, and the development of effective *Demodex* treatment strategies. Artificial skin models might also offer a promising approach for ex vivo *Demodex* cultivation. While a perfect human skin model remains elusive, the ability to cultivate *Demodex* hinges on replicating blood vessels and hair follicles. Initial research using human organotypic skin explant culture (hOSEC) shows promise in producing *Demodex* mites ex vivo [230].
- *Demodex* mite genetics require further study. Their tough chitinous exoskeletons pose a challenge for molecular research. Efforts to identify their complete genome have only recently begun [231, 232]. Recently, the genomic sequence of *Demodex folliculorum* was just unveiled [7]. This innovative study offers important new information on the evolutionary background and adaptations of *Demodex* mites. The article explores the evolutionary shift of *Demodex* mites from harmful ectoparasites to potentially commensal symbionts and the resulting genetic reduction that occurred during this process. The genome sequencing of *D. folliculorum* offers a useful tool for comprehending the evolutionary mechanisms influencing parasite-host relationships. It also clarifies how these small animals have adapted to their particular environment. The investigation is still in progress, and further research is required to completely comprehend the ramifications of this important finding. The cytochrome oxidase I (*cox1*) gene region offers a valuable tool for differentiating *Demodex* populations and might lead to reclassification of *D. folliculorum* due to potential polymorphisms [3, 231, 233]. Investigating these polymorphisms and their link to clinical presentations and epidemiology is crucial.

#### Future directions of diagnostic research

Diagnosing demodicosis remains a complex undertaking. While microscopic examination of skin scrapings (SSSB) is the current standard, it has limitations. The SSSB-determined threshold of 5 mites/cm^2^ for abnormality might not be universally applicable across all demographic groups and studies often lack control groups [32, 234]. Furthermore, the clinical significance of mites expelled by SSSB from deep follicles is unclear. Emerging techniques like dermatoscopy, confocal laser scanning microscopy, and high-definition optical coherence tomography show promise, but require further investigation regarding accuracy, reliability, and practicality in clinical settings [203,235,336]. Fluorescent staining combined with imaging holds potential for rapid and accurate mite detection, but confirmation is needed [237]. Additionally, a consensus regarding the clinical diagnostic criteria of demodicosis must be established and verified. A preliminary diagnostic criteria that has been proposed and is to be further evaluated involves the following: (i) no history of concurrent or pre-existing inflammatory dermatoses, such as rosacea, acne, or perioral dermatitis; (ii) an unusual increase in mite number, which should be detected from the active lesions at the time of examination; and (iii) the disease only goes into remission following a sufficient therapeutic trial leading to a return to normal levels of *Demodex* mites using topical or systemic acaricides, but not with antibacterials with anti-inflammatory properties like doxycycline and tetracycline or macrolides [6, 213]. When evaluating these criteria, it is crucial to remember that diagnosing a disease caused by *Demodex* mites should not rely solely on an abnormal density of these mites in the affected skin area. It should also include the presence of skin lesions that can be linked to or explained by the action of *Demodex* mites. Enhanced diagnostics by developing of non-invasive, rapid and cost-effective diagnostic tools and establishing consensus on diagnostic criteria will improve early detection and accurate diagnosis, especially in routine clinical practice.

#### Future directions of therapeutic research

Despite the availability of various treatment options and the first FDA-approved treatment for *Demodex* blepharitis, XDEMVY™, there is no single, universally acknowledged therapy for Demodicosis. Treatment selection is individualized based on clinical presentation and underlying conditions. However, there is little robust evidence-based research on treatment alternatives [213]. Key questions and research gaps in demodicosis treatment include:

- *Role of anti-inflammatory medications*: It remains unclear whether the effectiveness of tetracyclines, macrolides, azelaic acid, or metronidazole in *Demodex*-associated conditions like rosacea is primarily due to their anti-inflammatory properties or also includes acaricidal effects. Verification of the theory that tetracyclines target *Demodex* mites via their endosymbiotic bacteria, *B. oleronius*, is necessary [81]. The optimal dosage of metronidazole for demodicosis compared to ivermectin also requires investigation.
- *Evaluation of additional acaricides*: While approved for scabies, the efficacy of topical acaricides like benzyl benzoate, crotamiton, lindane, or malathion in demodicosis treatment is largely unknown. Further research in this area is warranted [238].
- *Moxidectin for human demodicosis*: Exploring the potential of moxidectin, currently used for canine demodicosis and human onchocerciasis, for topical treatment of human demodicosis is an interesting avenue for future work [239].
- *Acaricide resistance and recurrence*: Research on the emergence of acaricidal resistance in *Demodex* mites and the mechanisms of mite repopulation after acaricide treatment is entirely lacking [40]. Verifying the efficacy of combination therapies in preventing recurrence requires the development of in vitro human mite cultures to identify the most effective treatment strategies [3].
- *Alternative therapies*: While the potential of plant-derived preparations like essential oils for demodicosis treatment is being explored, more in vivo studies are needed to assess their efficacy [240]. Investigating the role of auxiliary chemicals within these preparations in influencing their effectiveness is also important [241].
- *Research on specific botanicals*: Further research is necessary to evaluate promising botanical preparations like clove oil, ginger oil, camphor oil, bergamot oil, peppermint oil, salvia oil, black seed oil, medicinal alpine rhizome, prickly pear peel oil, orange fruit oil, and cinnamon bark oil for Demodicosis treatment [240].
- *Personalized treatment in patients with different immune status*: There is a significant research gap in the area of personalized *Demodex* treatment for patients with a compromised immune systems. Developing evidence-based protocols is hampered by the paucity of clinical trials in this population, the variability of immunosuppression status, and possible drug interactions with the multiple medications taken for the underlying conditions. More research is needed to determine how particular immunodeficiencies affect *Demodex* presentation and treatment response. It is yet unknown how long treatments should last, what regimens are best, and what role adjunctive therapy such as local corticosteroids should play. Investigating alternate therapies is necessary due to worries about the emergence of resistance in those patients. It is imperative that these gaps be filled by focused research in order to improve outcomes for this vulnerable population [2, 4, 84].

In conclusion, accurate diagnosis and effective treatment of demodicosis are essential to prevent the social and aesthetic burdens associated with this condition [242]. Future research should focus on refining diagnostic techniques, evaluating existing and novel treatment options, and understanding the mechanisms of mite repopulation and potential acaricide resistance.

### Improving public health management of *Demodex* mite infections

A multifaceted strategy using a range of implementation methods is necessary for the effective public health management of *Demodex* mite infections. These may include the following specific tips [2, 243–247]:

1. Increased public awareness and health education:

• Information Distribution: To increase public knowledge of* Demodex* mites, their possible effects, and risk factors, targeted public health campaigns using a variety of channels (such as social media, professional societies networks, and community outreach initiatives) are essential.

• Educational materials: Creating and disseminating easily comprehensible educational materials for the general public and healthcare professionals, such as booklets, pamphlets, and internet resources.

• School health programs: To raise awareness among young students, provide age-appropriate information about skin care and *Demodex* mites in school health educational syllabi.

In another section of this manuscript, further information on raising awareness among patients, healthcare providers, and other caregivers is provided below.

2. Early diagnosis and screening:

• Better diagnostic methods: Ongoing research and development of more accessible and sensitive diagnostic instruments, such as dermatoscopy and other non-invasive techniques, for identifying *Demodex* mites.

• Early detection programs: To enable early diagnosis and treatment, specific screening programs are to be implemented for high-risk groups (such as those with rosacea, blepharitis, or compromised immune systems).

• Training of healthcare professionals: Educating medical professionals (primary care doctors, dermatologists, and ophthalmologists) on how to identify the symptoms of illnesses linked to *Demodex* and how to use the right diagnostic tests in their clinical practice. Training programs are crucial to ensuring that clinicians possess the necessary knowledge, skills, and support to diagnose various forms of the *Demodex*-related diseases in different patients' groups. In a subsequent section of this review, comprehensive details regarding healthcare professional training are included.

3. Development and research:

• Innovative therapeutic approaches: To effectively treat *Demodex* mite infestations, ongoing research and development of innovative therapeutic interventions, including as topical and oral drugs, and possibly even biological therapies, is necessary.

• Pathogenesis investigation: More research is needed to understand the exact mechanisms by which *Demodex* mites cause skin conditions and to find possible targets for treatment.

• Epidemiological studies: To gain a better understanding of the prevalence, distribution, and risk factors linked to *Demodex* mite infections in various populations, epidemiological studies are important.

Other sections of this review included more details on research needs in a in a variety of *Demodex*-related fields, including parasite biology, pathogenesis, diagnosis, treatment, prevention, and control.

4. Collaboration and arrangement:

• Multidisciplinary collaboration: To promote information sharing, resource sharing, and the creation of integrated management plans, dermatologists, ophthalmologists, parasitologists, public health officials, and researchers should collaborate.

• Public-private partnerships: Using collaborations among pharmaceutical corporations, research institutes, patient advocacy organizations, and public health authorities to expedite research, expand access to care, and improve public health outcomes.

By putting these tactics into practice, we may greatly enhance the public health management of *Demodex* mite infections, reducing their impact on individual health and well-being while mitigating their potential public health consequences.

### Improving clinicians' awareness and public education

Healthcare professionals, particularly dermatologists, need to become more aware of Demodicosis, a challenging condition that mimics other skin and eye diseases. Recognizing *Demodex* mites as a potential cause in various presentations would lead to earlier diagnosis, appropriate treatment, and improved patient outcomes [2]. Dermatologists and ophthalmologists should incorporate parasitological analysis alongside bacteriological tests in their diagnostic routines [84]. Conventional treatments for suspected skin and eye conditions like acne, rosacea, and blepharitis that fail to improve may warrant investigation for *Demodex* infestation [85]. Similarly, it is likely essential to consider the possibility of demodicosis when diagnosing a single or multiple facial skin lesions in patients with compromised immune systems or those undergoing immunosuppression. This is especially true given the low cost and ease of use of the *Demodex* test. It results in the initiation of the proper treatment and an improvement in the skin's status [4]. Patients should also be assessed for demodicosis if they have a history of long-term improper topical steroid use on their faces and exhibit clinical signs of either steroid-induced dermatitis or steroid addiction dermatitis [129, 130]. When treating *Demodex* in immunocompromised patients, the patient's specific immunodeficiency, possible drug interactions, the severity of the disease, and the risk of aggravating underlying conditions or secondary infections must all be carefully considered. The response to treatment may change in those patients, requiring close monitoring and possibly long-term management with systemic plus topical therapies [4]. Patient-specific factors, such as age and pregnancy, and specialist collaboration are also essential for maximizing outcomes. While there are currently no universally "approved" guidelines dedicated specifically to the treatment of *Demodex* exclusively for immunocompromised individuals, given the variability of immunocompromised conditions and the need for individualized treatment, the following outlines the most significant literature-based best practices for treating *Demodex* infections in immunocompromised patients, emphasizing the personalized approach [2, 4, 84]:

•Individualized treatment: Tailoring therapy based on the patient's specific immune status, infestation severity, and potential drug interactions.

•Combination therapy: Applying both local and systemic therapies, particularly for situations that are severe or persistent.

•Ivermectin as a core treatment: Relying on ivermectin either oral or topical, with oral ivermectin being saved for cases with severe immunosuppression.

•Cautious use of topical acaricides: Using products like permethrin, metronidazole, and tea tree oil while being aware of the possibility of skin irritation.

•Rigorous monitoring: Paying close observation to patients' responses to treatment and any adverse reactions.

In conclusion, the management of *Demodex* mite diseases necessitates a personalized approach that takes into account the patient's immune status, clinical presentation, and individual circumstances [2, 4, 84].

Comprehensive training programs are essential for healthcare professionals to effectively address health issues associated with *Demodex* mites and end the neglect of *Demodex*-related diseases. These programs ensure that clinicians have access to the latest information, best practices, and support they need to provide high-quality care to their patients. Training modalities should be diverse and may include conferences, symposiums, workshops, on-the-job training sessions, and continuing education programs. The following training methods and tools can be implemented [244, 248, 249]:

• Conferences and symposiums: provide opportunities for knowledge sharing, presentations on new research, and discussions on emerging diseases like *Demodex* mite infections.

• Published journals and newsletters: These publications disseminate research findings, clinical updates, and best practices to healthcare providers.

• Developing and maintaining professional guidelines: These guidelines provide evidence-based recommendations for the diagnosis and management of various health conditions related to *Demodex* mites.

• Continuing medical education (CME) programs: These programs offer opportunities for healthcare providers to update their knowledge and skills. They can take various forms, such as online courses: Interactive modules and webinars that can be accessed remotely.

• In-person workshops: Hands-on training sessions and lectures conducted by experts.

• Grand rounds: Presentations and discussions of interesting or challenging cases within a hospital or clinic setting.

The cornerstone of demodicosis prevention lies in education. Patients, caregivers, and at-risk individuals should be informed that controlled *Demodex* mites residing within hair follicles are harmless. Regular cleansing of the face and eye area with soaps, shampoos, and wipes plays a vital role in preventing overgrowth. Frequent washing of household linens and clothes at high temperatures further helps to eradicate parasites [34]. While patient education on good hygiene is paramount, extending hygiene instruction to staff in beauty salons would also contribute to prevention [179]. Simple measures like twice-daily cleansing with non-soap cleansers, avoiding oily cosmetics and cleansers, and regular exfoliation to remove dead skin cells can significantly reduce the risk of excessive *Demodex* mite proliferation [84].

## Conclusion

*Demodex* mites, once considered skin commensals, have emerged as significant players in human skin and eye health and disease. Demodicosis, the pathological condition resulting from infestation by these parasitic arachnids, has been historically understudied and underdiagnosed. The disease remains overshadowed by other skin diseases, hindering research and development of effective diagnostic and therapeutic tools. This neglect has significant consequences for both individual and public health. A paradigm shift is necessary in our understanding and management of this ubiquitous ectoparasite and the illnesses it causes. The current neglect of demodicosis requires urgent attention.

### Challenges in combating neglect

- *Diagnostic ambiguity* Demodicosis diagnosis remains a hurdle due to overlapping symptoms with other skin conditions. Standardized diagnostic methods and agreed-upon criteria for *Demodex* visualization and enumeration are lacking.
- *Limited standard therapeutic options* Current therapies are primarily repurposed drugs with variable effectiveness and long-term safety and resistance concerns. Without a single, universally accepted treatment, more efficacious and tolerable options need to be developed.
- *Scarcity of research funding* Limited research funding, possibly due to the perceived cosmetic nature of the disease and the lack of commercially viable therapeutic targets, hinders our understanding of *Demodex* biology, host-parasite interactions, and novel treatment strategies. Our current understanding of human demodicosis relies heavily on individual case reports, and lacks robust empirical evidence.

**Fig. 5:**
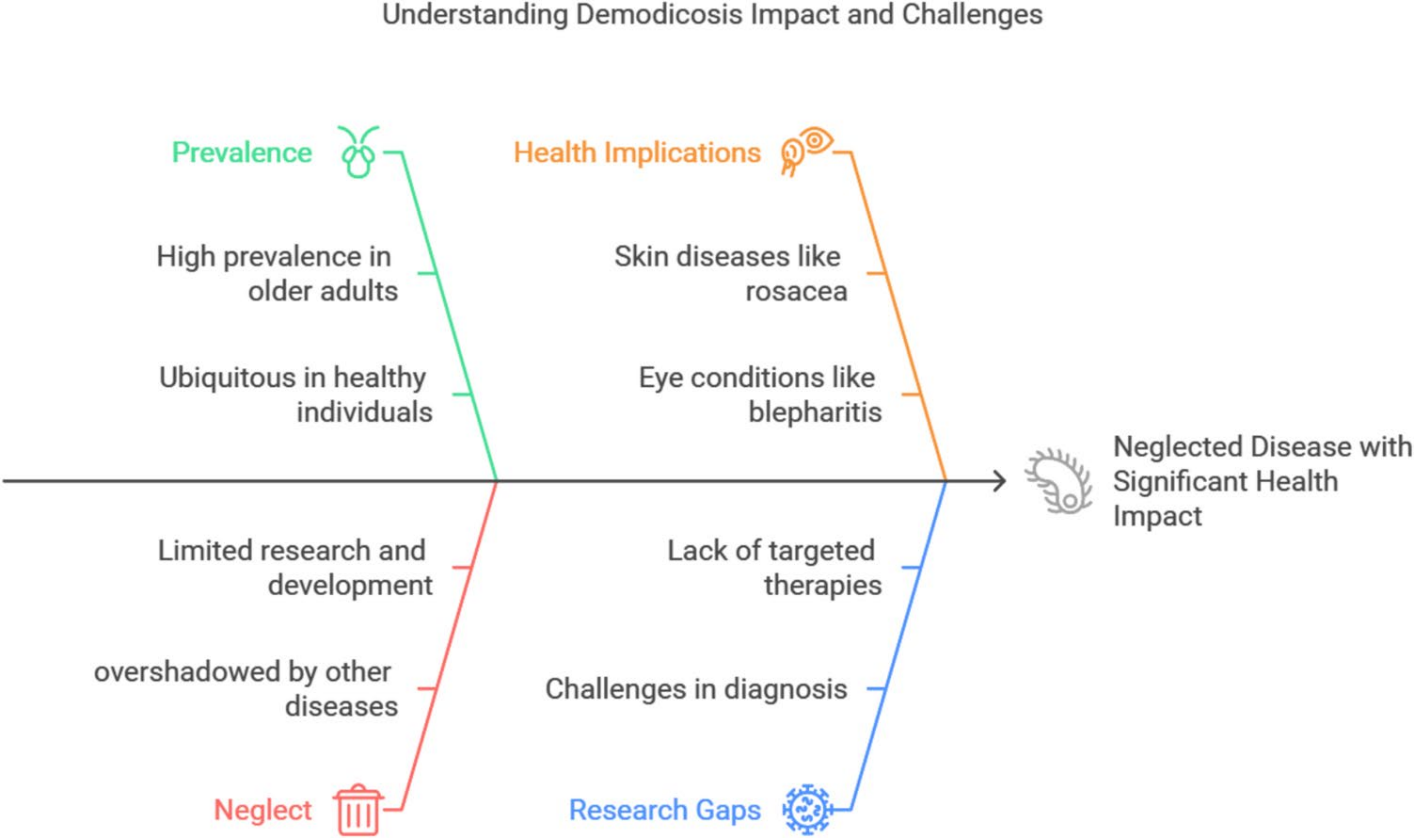
Understanding demodicosis impact and challenge

Figure 5 summarizes the impact and challenges of *Demodex* mites and their linked diseases.

### Future prospects for eradicating neglect

*•Enhanced diagnostics—*Developing non-invasive, rapid, and cost-effective diagnostic tools alongside standardized diagnostic criteria will significantly improve early detection and accurate diagnosis in clinical practice. New techniques like dermatoscopy and imaging/fluorescence scanning show promise for rapid and precise mite detection and quantification, but require further validation of their accuracy and practicality.

*• Enhanced understanding of the mite biology and pathogenesis*—Research on the mite-microbiome relationships, host immune response, life cycle, and susceptibility factors will unveil crucial insights into disease development and progression, potentially leading to personalized treatment strategies.

•*Targeted therapeutics*—Targeted therapeutics focused on disrupting mite-host interactions, modulating host immunity, regulating the microbiome, and specifically on the *Demodex* life cycle hold promise for more effective, long-lasting, and individualized therapies. Exploring other acaricides effective against different mites and the potential of natural products like plant-derived oils present exciting avenues for new treatment development. Establishing an ex vivo culture system is crucial to empirically assess the efficacy of potential therapies.

•*Increased awareness and research*—Raising public and scientific awareness and educating healthcare professionals about demodicosis is paramount. Advocating for its inclusion in public health programs and prioritizing research funding will drive further discoveries and advancements in diagnosis, treatment, and prevention, potentially leading to a significant public health impact. Continued research is essential to address knowledge gaps and develop better implementation strategies.

**Fig. 6:**
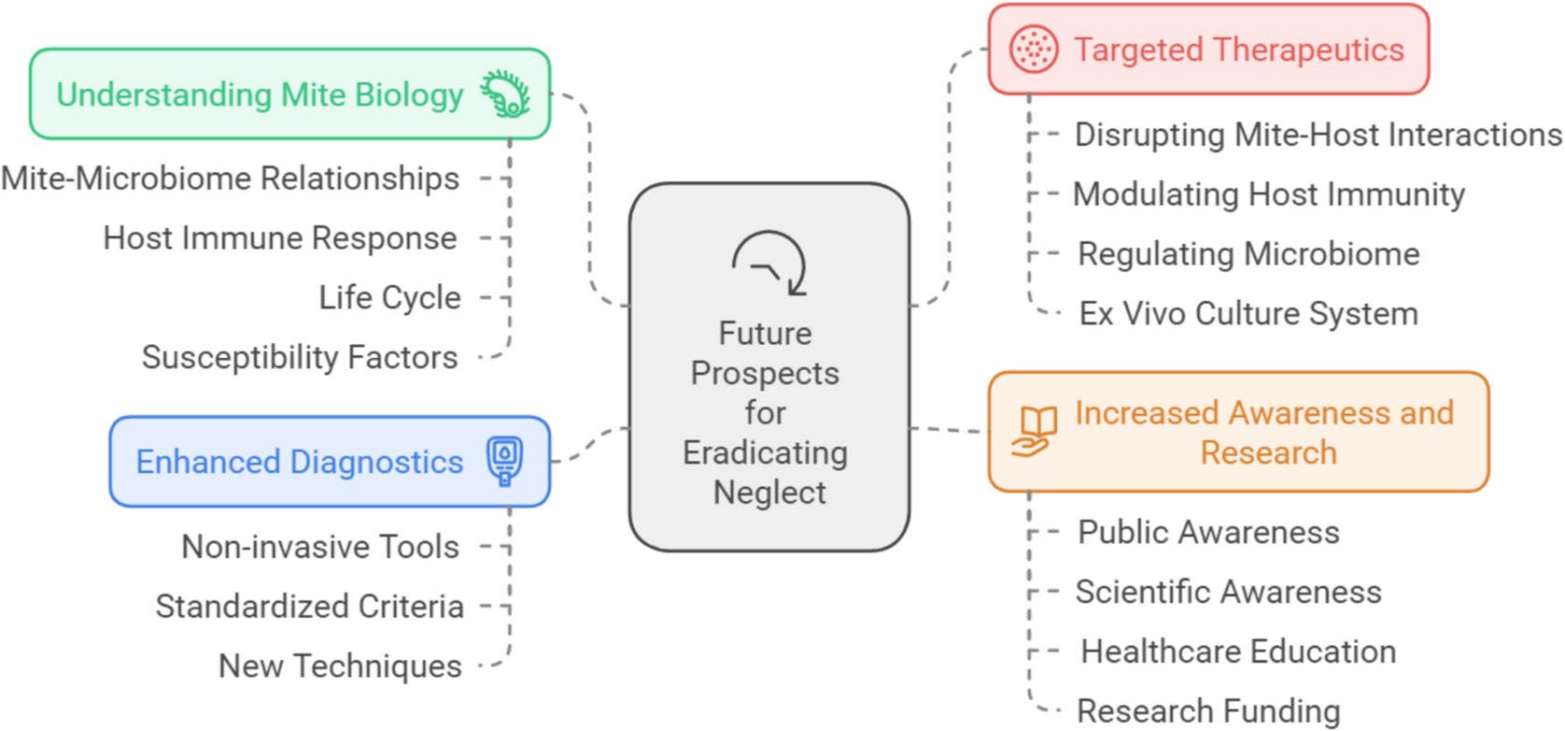
Future prospects for eradicating neglect of *Demodex* mites and their related diseases

Figure 6 summarizes the future prospects for eradicating neglect of *Demodex* mites and their associated diseases.

### Concluding words

*Demodex* mites and demodicosis have long been neglected. To better understand *Demodex* and treat demodicosis requires a multidisciplinary approach. It is critical to recognize the significant impact of this disease and address the current challenges through the collaborative efforts of researchers, clinicians, public health professionals, and policy makers. By investing in research, driving awareness, implementing advanced diagnostics, and developing targeted therapies, we could shed more light on this neglected disease, improve the lives of individuals suffering from it and ensure that they receive proper care, and ultimately bring the disease under control.

## Data Availability

No datasets were generated or analysed during the current study

## Abbreviations

HLA: Human Leukocyte Antigen
TLRs: Toll-like receptors
DLE: Discoid lupus erythematosus
BCC: Basal cell carcinoma
IGF-2: Insulin-like growth factor 2
LMDF: Lupus Miliaris Disseminatus Faciei
MGD: Meibomian gland dysfunction
SSSB: Standardized Skin Surface Biopsy
DME: Direct Microscopic Examination
SNS: Superficial Needle Scraping
RCM: Reflectance Confocal Microscopy
CLSM: Confocal Laser Scanning Microscopy
hOSEC: Human organotypic skin explant culture
cox1: Cytochrome oxidase I
IgD: Immunoglobulin deposition
PPR: Papulopustular rosacea
GABA-Cl: Gamma-Aminobutyric Acid- activated Chloride
